# Supplementary material for: Regulation of Yki/Yap subcellular localization and Hpo signaling by a nuclear kinase PRP4K
Source: Nat Commun. 2018 Apr 25;9:1657. doi: 10.1038/s41467-018-04090-2 (PMC5916879; doi:10.1038/s41467-018-04090-2)
Supplement: Supplementary file 1 — Supplementary Information [file 41467_2018_4090_MOESM1_ESM.pdf]

Supplementary Information

Regulation of Yki/Yap subcellular localization and Hpo signaling by a nuclear kinase PRP4K

Cho et al.

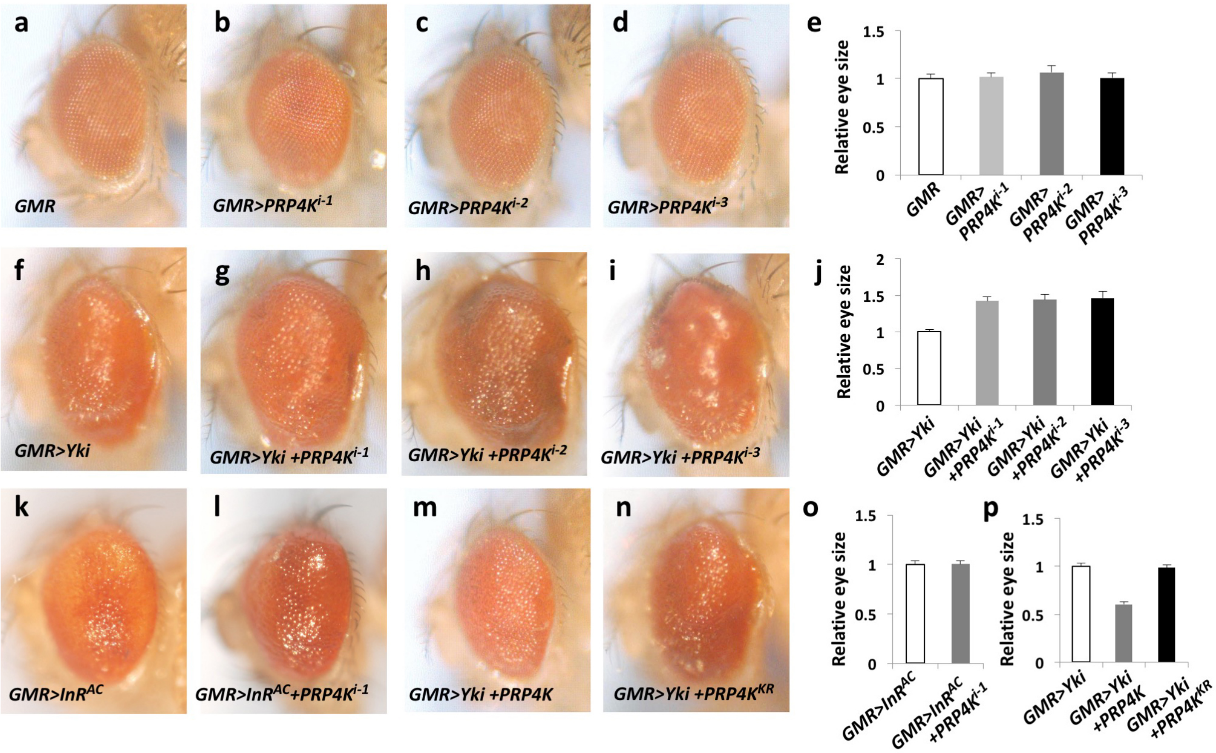

Supplementary Figure 1. PRP4K regulates organ size specifically through the Hpo pathway. (**a-d**) *Drosophila* adult eyes expressing *GMR-Gal4* alone (**a**) or in conjunction with the indicated *UAS-PRP4K-RNAi* lines (**b-d**). (**f-i**) *Drosophila* adult eyes expressing *GMR-Yki* alone (**f**) or in conjunction with the indicated *UAS-PRP4K-RNAi* lines (**g-i**). (**k, l**) *Drosophila* adult eyes expressing *GMR>InR*^{AC} alone (**k**) or in conjunction with *UAS-PRP4K-RNAi-1* (**l**). (**m, n**) *Drosophila* adult eyes expressing *GMR-Yki* together with *UAS-PRP4K* (**m**) or *UAS-PRP4K*^{KR} (**n**). (**e**, **j**, **o**, **p**) Quantification of eye size for the indicated genotypes. Data are means ± s.d. from three independent experiments. N≥5 for each genotype.

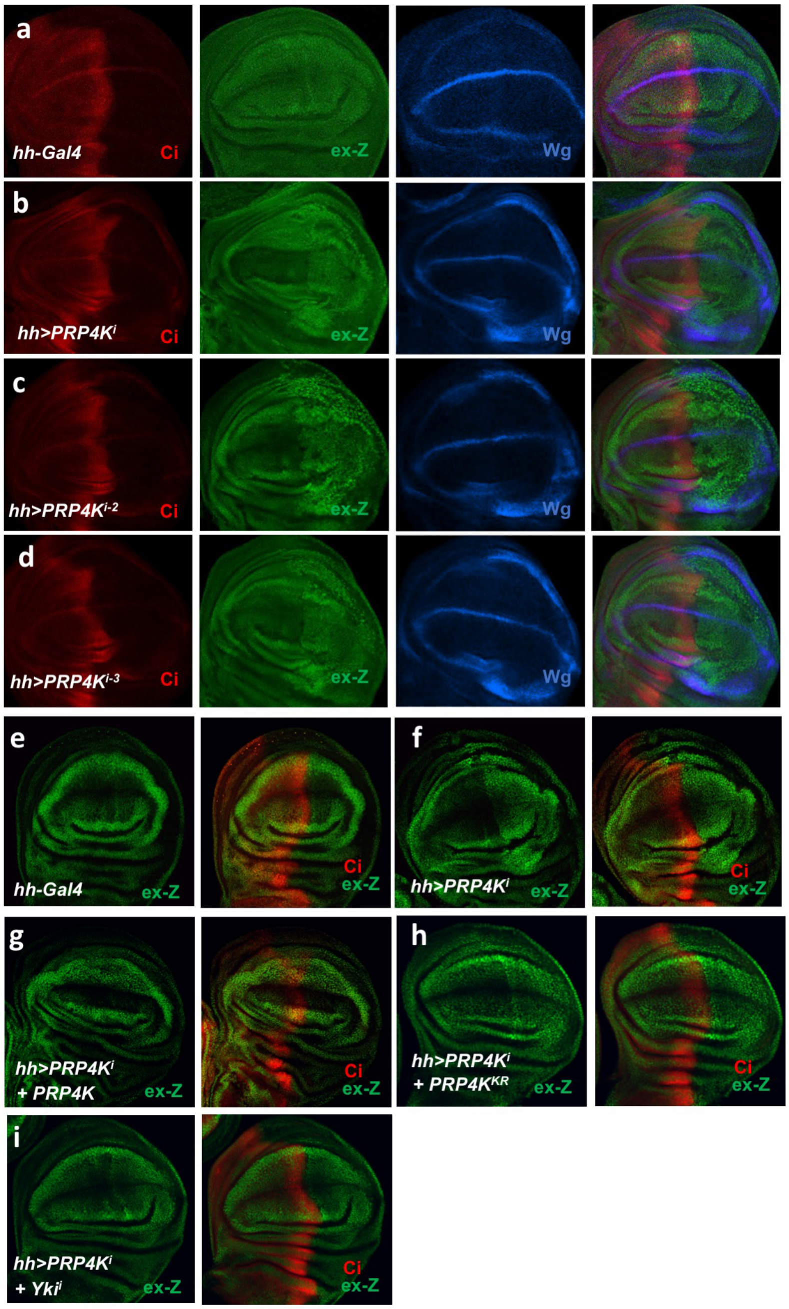

Supplementary Figure 2. PRP4K regulates the expression of Hpo target genes (**a-d**) Late third instar wing imaginal discs expressing *hh-Gal4* alone (**a**) or in conjunction with the indicated *UAS-PRP4K-RNAi* lines (**b-d**) were immunostained to show the expression of Ci (red), *ex-lacZ* (green), and Wg (blue). (**e-i**) Late third instar wing imaginal discs expressing *hh-Gal4* (**e**), *hh-Gal4/UAS-PRP4K-RNAi-1*(**f**), *hh-Gal4/UAS-PRP4K-RNAi-1 + UAS-PRP4K* (**g**), *hh-Gal4/UAS-PRP4K-RNAi-1 + UAS-PRP4K^{KR}* (**h**), or *hh-Gal4/UAS-Yki-RNAi* (**i**) were immunostained to show the expression of Ci (red) and *ex-lacZ* (green). Ci expression marks the anterior compartment.

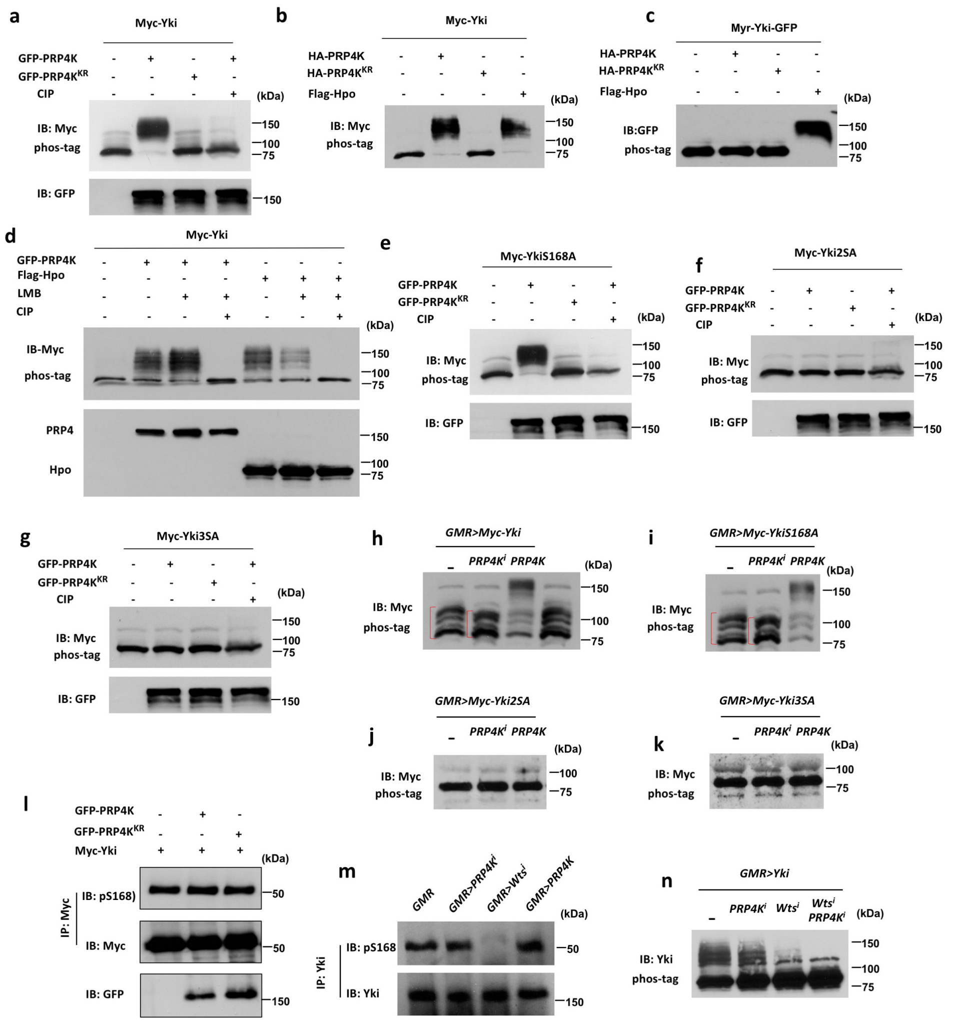

Supplementary Figure 3. PRP4K phosphorylates Yki at a subset of Wts sites. (**a**) PRP4K but not PRP4K^{KR} induced a mobility shift of cotransfected Myc-Yki on phos-tag gel, and the mobility shift was abolished by treating the S2 cell extracts with CIP. (**b, c**) PRP4K induced a mobility shift of Myc-Yki but not of Myr-Yki-GFP on phos-tag gel. By contrast, Hpo induced a mobility shift of both Myc-Yki and Myr-Yki-GFP on phos-tag gel. (**d**) Treating S2 cells with LMB increased PRP4K-mediated but decreased Hpo-mediated phosphorylation of Myc-Yki. (**e-f**) PRP4K but not PRP4K^{KR} induced phosphorylation of Myc-YkiS168A in S2 cells (**e**); however, PRP4K failed to induce phosphorylation of Myc-Yki2SA (**f**) or Myc-Yki3SA (**g**) as indicated by the lack of mobility shift on phos-tag gels. (**h-k**) Cell extracts made from late third instar eye discs expressing *GMR>Myc-Yki* (**h**), *GMR>Myc-YkiS168A* (**i**), *GMR>Myc-Yki2SA* (**j**), or *GMR>Myc-Yki3SA* (**k**) either alone, or in conjunction with *UAS-PRP4K-RNAi* or *UAS-PRP4K* were separated on phos-tag gels and immunoblotted with anti-Myc antibody. (**l**). Cells were transfected with the indicated constructs, and lysates were immunoprecipitated with a Myc antibody, followed by western blot analysis p-S168 and Myc antibodies, or directly subjected to western blot analysis with a GFP antibody. (**m**) Cell extracts made from late third instar eye discs expressing *GMR-Gal4*, *GMR-Gal4/UAS-PRP4K-RNAi*, *GMR-Gal4/UAS-Wts-RNAi,* or *GMR-Gal4/UAS-PRP4K* were immunoprecipitated with Yki antibody, followed by immunoblotting with p-S168 and Myc antibodies. (**n**) Cell extracts made from late third instar eye discs of the indicated genotypes were separated on phos-tag gels and immunoblotted with anti-Yki antibody.

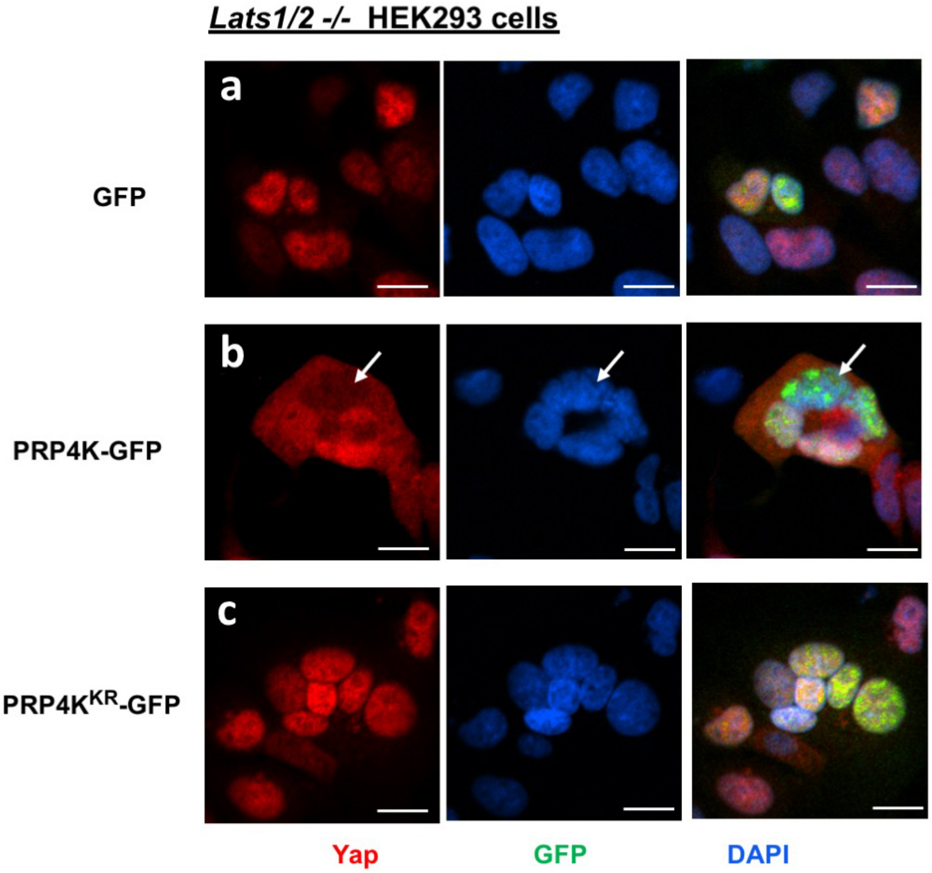

Supplementary Figure 4. Overexpression of PRP4K can inhibit Yap nuclear localization in the absence of Lats1/2. (**a-c**) Lat1/2 KO HEK293 cells transfected with GPF (**a**), GFP-PRP4K (**b**), or GFP-PRP4K^{KR} (**c**) expression constructs were stained with Yap and GFP antibodies and DAPI. Arrows indicate cells expressing PRP4K-GFP and exhibiting cytoplasmic localization of Yap. Scale bars, 10 μm.

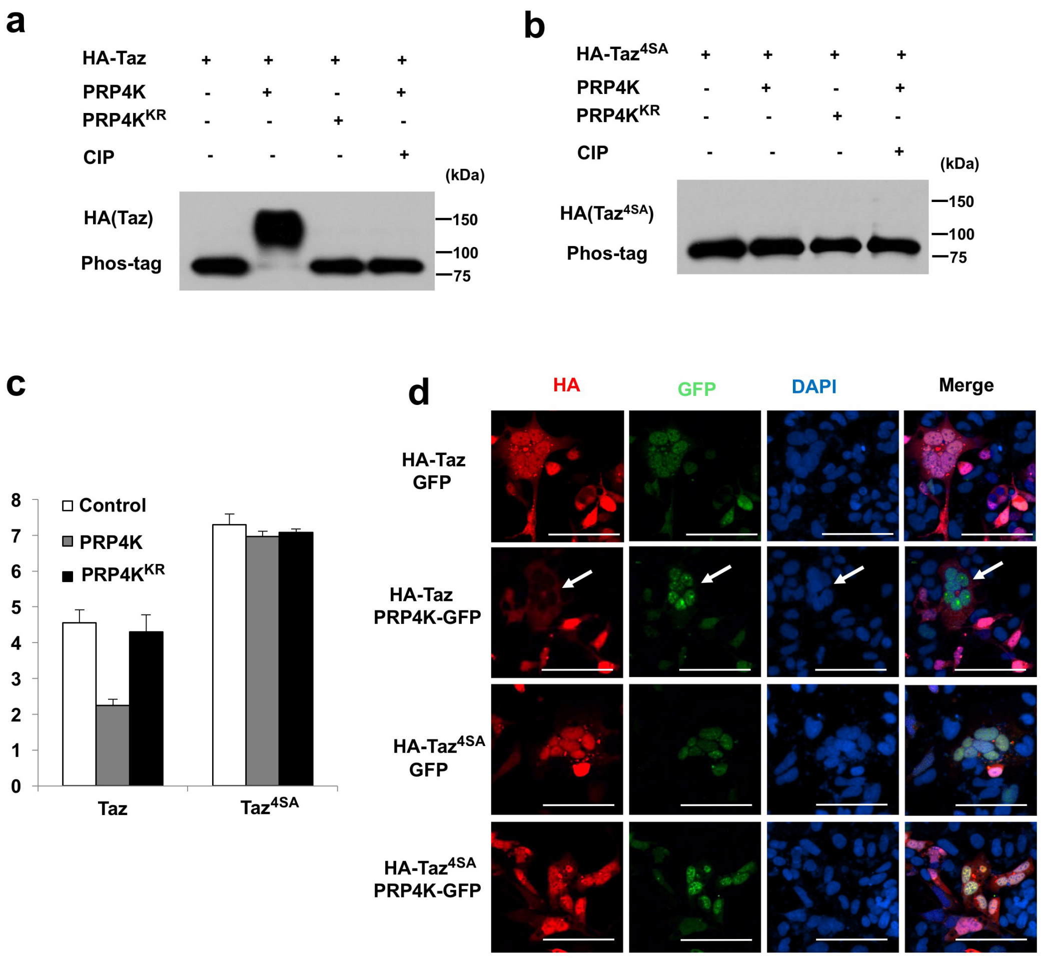

Supplementary Figure 5. PRP4 phosphorylates and inhibits TAZ. (**a**, **b**) Western blot analysis of cell lysates derived from HEK293A cells transfected with the indicated constructs. (**c**) *3XSd2-luc* reporter assay of HEK293A cells transfected with the indicated constructs. Data are means ± s.d. from three independent experiments. (**d**) HEK293A cells transfected with the indicated constructs were staining for HA, GFP and DAPI. Arrows indicate cells expressing PRP4K-GFP and exhibiting cytoplasmic localization of HA-Taz. Scale bars,100 μm.

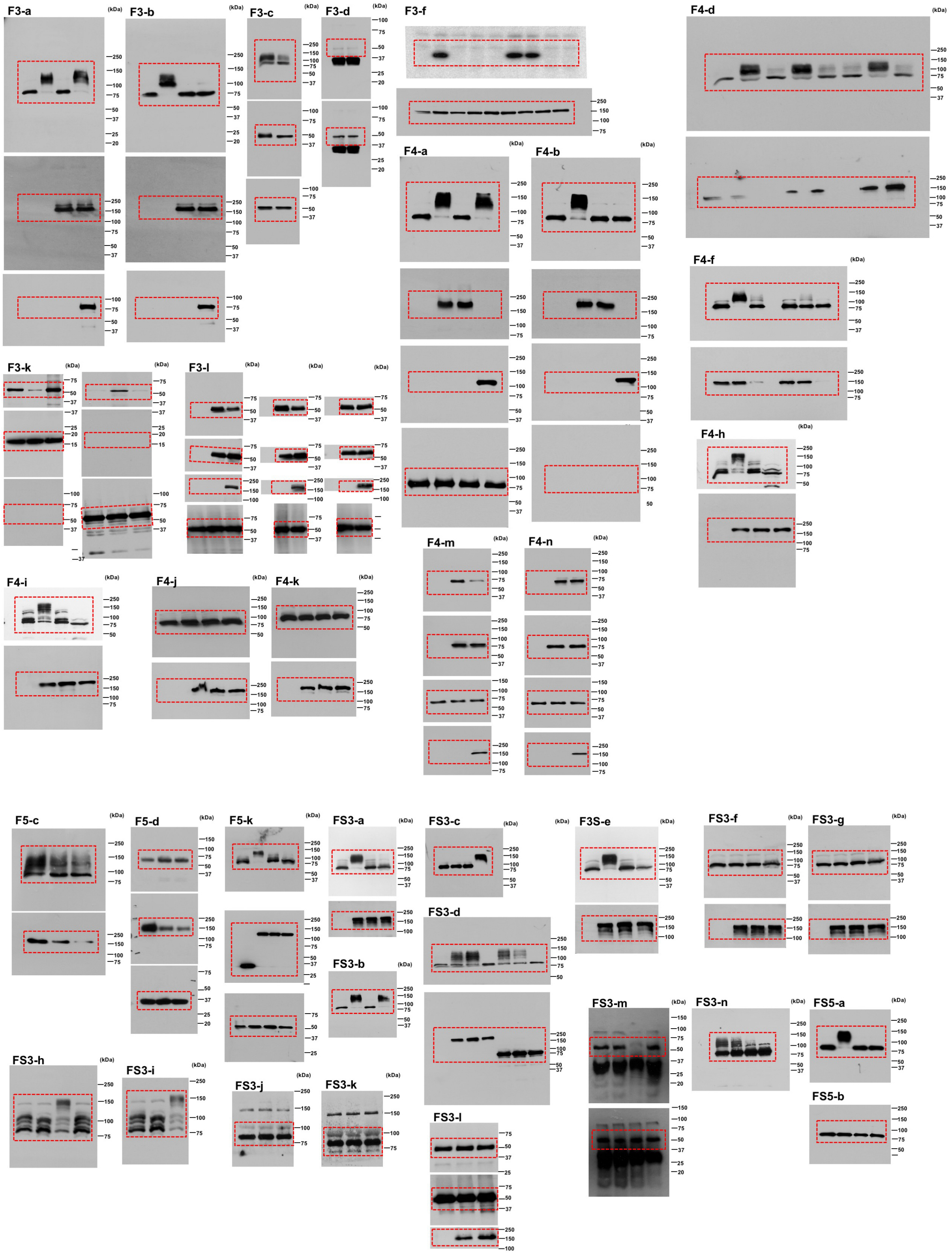

Supplementary Figure 9. Uncropped scans of blots shown in Fig. 3, Fig. 4, Fig. 5, supplementary Fig. 3, and supplementary Fig. 5
